# Decreased binding capacity (*B*_max_) of muscarinic acetylcholine receptors in fibroblasts from boys with attention-deficit/hyperactivity disorder (ADHD)

**DOI:** 10.1007/s12402-013-0103-0

**Published:** 2013-02-07

**Authors:** Jessica Johansson, Magnus Landgren, Elisabeth Fernell, Tommy Lewander, Nikolaos Venizelos

**Affiliations:** 1 Department of Clinical Medicine, School of Health and Medical Sciences, Örebro University, 701 82 Örebro, Sweden; 2 Unit of Neurodevelopmental Disorders, Department of Paediatrics, Skaraborg Hospital, 542 24 Mariestad, Sweden; 3 The Research and Development Centre, Skaraborg Hospital, 541 85 Skövde, Sweden; 4 The Gillberg Neuoropsychiatry Centre, Sahlgrenska Academy, Gothenburg, Sweden; 5 Department of Neuroscience, Psychiatry, Uppsala University Hospital, 750 17 Uppsala, Sweden

**Keywords:** ADHD, Muscarinic acetylcholine receptors, Receptor binding assay, Fibroblasts, Biomarker

## Abstract

Monoaminergic dysregulation is implicated in attention-deficit/hyperactivity disorder (ADHD), and methylphenidate and amphetamines are the most frequently prescribed pharmacological agents for treating ADHD. However, it has recently been proposed that the core symptoms of the disorder might be due to an imbalance between monoaminergic and cholinergic systems. In this study, we used fibroblast cell homogenates from boys with and without ADHD as an extraneural cell model to examine the cholinergic receptor density, that is, muscarinic acetylcholine receptors (mAChRs). We found that the binding capacity (*B*_max_) of [^3^H] Quinuclidinyl benzilate (^3^H-QNB) to mAChRs was decreased by almost 50 % in the children with ADHD (mean = 30.6 fmol/mg protein, SD = 25.6) in comparison with controls [mean = 63.1 fmol/mg protein, SD = 20.5, *p* ≤ 0.01 (Student’s unpaired *t* test)]. The decreased *B*_max_ indicates a reduced cholinergic receptor density, which might constitute a biomarker for ADHD. However, these preliminary findings need to be replicated in larger ADHD and comparison cohorts.

## Introduction

Attention-deficit/hyperactivity disorder (ADHD) is the most common neurodevelopmental disorder, with a worldwide prevalence of about 5–7 % in childhood (Faraone et al. 2003; Polanczyk et al. 2007). The catecholaminergic systems are implicated in the pathophysiology of ADHD, and the pharmacological treatment for ADHD includes central stimulants that enhance the neurotransmission of dopamine and norepinephrine through multiple actions. Interactions between the dopaminergic and the serotonergic neurotransmitter systems suggest that serotonin, in addition to dopamine, is likely to be linked to the disorder (Oades 2008).

Recently, it was suggested that the core symptoms of ADHD might be attributable to an imbalance between the neuromodulatory effects of monoamines and acetylcholine (Ach), implicating a monoaminergic–muscarinic receptor interaction (Vakalopoulos 2007). ACh plays an important role in regulating central nervous system (CNS) functions, including memory, attention and motivation processes—all of which have been found to be dysregulated in ADHD. Improvement of ADHD symptoms has been reported in adults and youths treated with cholinergic enhancers, such as Ach partial agonists (e.g. ABT-089) and acetylcholinesterase (AChE) inhibitors (e.g. donepezil) (Doyle et al. 2006; Wilens et al. 2006), although results are inconclusive (Cubo et al. 2008). Atomoxetine, a non-stimulant drug used for treating ADHD has been found to increase cholinergic neurotransmission in animals, indicating a procholinergic profile of this drug (Tzavara et al. 2006).

With respect to ADHD neurotransmission research, there is a need for extraneural patient-derived cell models and biochemical markers that are detectable and quantifiable in easily accessible tissues, but still reflecting neuronal conditions. One such possible cell model is primary skin fibroblasts that are being used for investigating biochemical differences in various neuropsychiatric disorders, such as Parkinson’s disease, schizophrenia and Alzheimer’s disease (Auburger et al. 2012; Mahadik and Mukherjee 1996; Vestling et al. 1995). Recent studies have also demonstrated that fibroblast cells can be directly converted into functional neurons, which further can be directed towards distinct functional neurotransmitter phenotypes (Pfisterer et al. 2011; Vierbuchen et al. 2010).

Skin fibroblasts represent a model of primary human cells and comprise the polygenic risk factors of specific patients. This model has many advantages compared to other peripheral cell models (e.g. platelets and lymphocytes), one being that after several passages in culture, fibroblasts are free from state-related changes and effects of medications, drugs and hormones, and thus patients do not have to be unmedicated at the time of the biopsy collection (Mahadik and Mukherjee 1996).

Given the relevant impact of cholinergic dysregulation in ADHD, the aim of the present study was to examine the density of muscarinic acetylcholine receptors (mAChRs) in fibroblasts from children with ADHD, and in a comparison group, in an in vitro receptor binding assay.

## Materials and methods

The study was approved by the Regional Ethics Committee in Gothenburg, Sweden. A written and signed consent was obtained from the parent/s and their child before performing the studies.

### Subjects

The index group consisted of fibroblast cell lines from 11 boys with ADHD of the combined type (inattention combined with hyperactivity and impulsivity) according to the Diagnostic and Statistical Manual of Mental Disorders (DSM-IV) (APA 1994). They were between 10 and 11 years of age (mean 10.5 years) and treated with either methylphenidate or atomoxetine. They were consecutively recruited from a specialized paediatric unit for children and adolescents with ADHD at Skaraborg’s hospital in the south-western part of Sweden. With respect to aetiology, at least nine of the 11 boys had a clear hereditary origin, that is, had first-grade relatives with ADHD and uneventful pregnancy and perinatal periods. Children with intellectual disability or meeting full criteria for autism spectrum disorders were excluded from the study. The comparison group comprised cell lines from nine boys, between 7 and 12 years (mean 9 years), without any diagnosis of a neurodevelopmental disorder. This group was recruited in conjunction with surgery at the ear, nose and throat clinic at Skaraborg’s hospital.

### Cell culture

In order to prepare fibroblast cell homogenates to be used in the receptor binding assays, each cell line was seeded in T_75_ tissue culture flasks (Costar Europe Ltd, Costar NY, USA) containing minimal essential medium (MEM, supplemented with 10 % foetal bovine serum (FBS, Gibco^®^), l-glutamine (2 mM/L), penicillin (100 mg/mL), streptomycin (100 mg/mL) and Amino-Max™ (0.06 % vol/vol). All growth media, antibiotics and FBS were obtained from Invitrogen, Life Technologies Europe BV, Sweden. The cells were incubated at a temperature of 37 °C in the presence of 5 % CO_2_ and high humidity. When the cells reached confluence, they were harvested by trypsinization, washed twice with phosphate-buffered saline [PBS, National Veterinary Institute (SVA), Sweden] and diluted in 1.5 mL of sterile Milli Q water. Protein concentrations for each cell homogenate were determined by the Bradford method, using bovine serum albumin as a standard (approximately 1 mg/mL of cell protein per cell line was used in the receptor binding assay). The cell homogenates were stored at −20 °C until used in the receptor binding assay. Cell lines between 7th and 15th passages (i.e. the number of splitting) were used in the experiments.

### Receptor binding assay

The receptor binding assay in fibroblast cell homogenate was done according to the modified procedure of Vestling et al. (1995). This was done by adding 25 μL of different concentrations (i.e. 0.1–3.0 μM) of [^3^H] Quinuclidinyl benzilate (^3^H-QNB, specific activity 50.5 Ci/mmol, Larodan Fine Chemicals AB, Sweden) into plastic tubes, containing 200 μL sodium phosphate buffer (pH 7.4). Half of the tubes contained unlabelled atropine (1.0 × 10^−4^ μM, Sigma-Aldrich, USA) to correct for non-specific binding. Fibroblast cell homogenates (25 μL; protein concentrations approximately 1 mg/mL) were added to the tubes, making a total volume of 250 μL. Tubes were incubated at 22 °C for 60 min and filtered rapidly through Whatman GF/C glass fibre filters (Fisher Scientific, Sweden) presoaked for 50 min in 0.05 % polyethyleneimine solution. The filters were washed twice under vacuum with ice-cold sodium phosphate buffer. The filters were then placed in scintillation vials to which scintillation liquid was added (Optiphase Hisafe 3, PerkinElmer Life Science, USA) and the disintegrations per minute (DPM) was counted by a scintillation counter (Winspectral 1414, PerkinElmer Life Science, USA).

### Calculations and statistical analysis

The specific binding of ^3^H-QNB was calculated by subtracting the values for non-specific binding in the presence of unlabelled atropine from those of total binding in the absence of atropine. All incubations were performed in duplicates and mean values were used for the calculation of binding characteristics. The binding capacity (*B*_max_) and the equilibrium dissociation constant (*K*_D_) of mAChR antagonist QNB were determined by nonlinear regression analysis.

Assumptions about parametric methods were met for *K*_D_ of ADHD patients, but not for *K*_D_ of controls or for *B*_max_ of ADHD patients and controls (determined by the D′Agostino and Pearson omnibus normality test). Therefore, the nonparametric Mann–Whitney *U* test was used to compare the *B*_max_ and *K*_D_ values between patients and controls. However, three outliers were identified by the scaled Median Absolute Deviation (MAD_E_) method and by Tukey’s method; one in the comparison group and two in the ADHD group. If excluding these outliers, assumptions about parametric methods were met and Student’s unpaired *t* test was used to compare the *B*_max_ and *K*_D_ values between patients and controls. For all statistical analyses, a significant level of 5 % was accepted and all calculations and analyses were performed using GraphPad Prism version 5 for Windows.

## Results

**Fig. 1:**
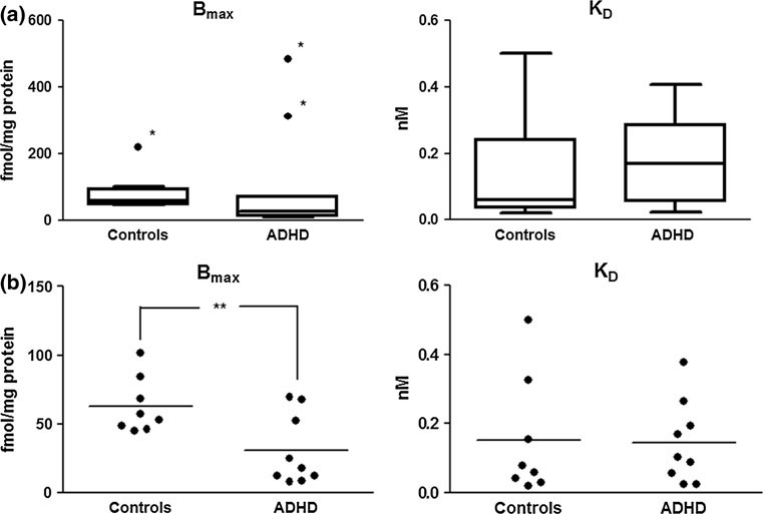
Binding characteristics of [^3^H] Quinuclidinyl benzilate (^3^H-QNB) binding to muscarinic acetylcholine receptors (mAChRs) in fibroblasts from children with attention-deficit/hyperactivity disorder (ADHD). **a** Illustrates the individual and median (*horizontal bars*) *B*_max_ and *K*_D_ values of mAChRs in fibroblasts from children with ADHD (*n* = 11) and controls (*n* = 9), including outliers. *Outliers, identified by the scaled Median Absolute Deviation (MAD_E_) method. **b** Illustrates the individual and mean (*horizontal bars*) *B*_max_ and *K*_D_ values of mAChRs in fibroblasts from children with ADHD (*n* = 9) and controls (*n* = 8), excluding outliers. *B*_max_ indicates binding capacity, *K*_D_ indicates the equilibrium dissociation constant and mAChRs indicates muscarinic cholinergic receptors. **p ≤ 0.01

Children with ADHD had a significantly lower binding capacity (*B*_max_) of the mAChR ligand QNB in comparison with controls (*p* ≤ 0.01) when excluding the three outliers identified by the MAD_E_ method. The *K*_D_ did not differ significantly between the two groups. These results suggest that the children with ADHD had a reduced density (~50 %) of mAChRs in fibroblasts, but that the affinity of ^3^H-QNB binding to the cholinergic receptors was not affected (Fig. 1).

## Discussion

The main finding in the present study was that children with ADHD had a significantly lower *B*_max_ of the mAChRs ligand QNB in comparison with controls. These results suggest that the children with ADHD had a reduced density of mAChRs in fibroblasts, which potentially could be due to genetic (e.g. mutation/s in the genes coding for mAChRs) and/or post-transcriptional (e.g. mRNA stability) factors. To our knowledge, similar findings have not been reported previously. However, three outliers were identified and we can only speculate about the reason for these extreme values, as they were not caused by experimental errors. The two ADHD outliers may represent different subgroups of ADHD and at least one of these boys had no evidence of hereditary origin. Since ADHD is a very heterogeneous disorder with regard to molecular genetics and phenotypic diversity (Thome et al. 2012), the finding may nevertheless be of relevance. However, further studies including different subgroups of ADHD are needed in order to draw firm conclusions.

We used fibroblast cells, derived from skin biopsies, obtained from children with ADHD and from controls, since these cells are considered to be a relevant experimental model for functional studies in humans (Stahl 1985; Auburger et al. 2012). Despite our finding of a difference in the density of mAChRs in fibroblasts of children with ADHD and controls, we can only speculate that the density of mAChRs is also decreased in the CNS. Until now, to our knowledge, no studies on mAChRs density have been performed in vivo in children with ADHD.

In a recent study, muscarinic cholinergic receptor binding (I-MR) was found to be lower (determined in lymphocytes) in children with ADHD (Coccini et al. 2009). This is in accordance with our findings; however, in that study, the I-MR was only significantly decreased in girls with ADHD, but not in boys. No explanation/theory for these gender differences was given.

We are aware of the limitations of our study, such as a small sample size, a small comparison group and the index group consisting of only boys. The identification of outliers would possibly be explained by the large heterogeneity and complexity of molecular factors underlying ADHD. Therefore, the results should be considered as preliminary. Future studies have to include larger sample size consisting of both boys and girls with hereditary and non-hereditary ADHD.

We conclude that fibroblasts derived from patients with ADHD offer a useful model for exploring neurological aspects in vitro, thus allowing new hypothesis and drugs to be tested. The indicated reduced density of mAChRs in fibroblasts from children with ADHD may constitute a biomarker for ADHD; however, these preliminary findings need to be replicated.
